# Studying Lipid Membrane Interactions of a Super-Cationic Peptide in Model Membranes and Living Bacteria

**DOI:** 10.3390/pharmaceutics14102191

**Published:** 2022-10-14

**Authors:** Isabel Pérez-Guillén, Òscar Domènech, Adrià Botet-Carreras, Alexandra Merlos, Josep M. Sierra, Fernando Albericio, Beatriz G. de la Torre, M. Teresa Montero, Miguel Viñas, Jordi H. Borrell

**Affiliations:** 1 Laboratory of Molecular Microbiology & Antimicrobials, Faculty of Medicine & Health Sciences, University of Barcelona, 08907 Barcelona, Spain; 2 Physical Chemistry Section, Faculty of Pharmacy and Food Sciences, University of Barcelona, 08028 Barcelona, Spain; 3 Institute of Nanoscience and Nanotechnology (IN2UB), University of Barcelona, 08028 Barcelona, Spain; 4 Peptide Sciences Laboratory, School of Chemistry and Physics, University of KwaZulu-Natal, University Road, Westville, Durban 4001, South Africa; 5 CIBER-BBN, Networking Centre on Bioengineering, Biomaterials and Nanomedicine, Department of Organic Chemistry, University of Barcelona, 08028 Barcelona, Spain; 6 KRISP, College of Health Sciences, University of KwaZulu-Natal, Westville, Durban 4001, South Africa

**Keywords:** super-cationic peptides, atomic force microscopy, anisotropy, antimicrobials interactions

## Abstract

The super-cationic peptide dendrimers (SCPD) family is a valuable class of antimicrobial peptide candidates for the future development of antibacterial agents against multidrug-resistant gram-negative bacteria. The deep knowledge of their mechanism of action is a major challenge in research, since it may be the basis for future modifications/optimizations. In this work we have explored the interaction between SCPD and membranes through biophysical and microbiological approaches in the case of the G1OLO-L_2_OL_2_ peptide. Results support the idea that the peptide is not only adsorbed or close to the surface of the membrane but associated/absorbed to some extent to the hydrophobic-hydrophilic region of the phospholipids. The presence of low concentrations of the peptide at the surface level is concomitant with destabilization of the cell integrity and this may contribute to osmotic stress, although other mechanisms of action cannot be ruled out.

## 1. Introduction

Bacteria are essential for human survival, but they can also cause severe and potentially fatal diseases [1]. One of the greatest achievements in medical history was the discovery of antibiotics and their clinical introduction to treat infections [2]. Nowadays, however, infections that were once treatable are no longer responsive to many antibiotics, due to the increase in antimicrobial resistance (AMR). Indeed, roughly 700,000 people die every year due to drug-resistant infections, with predictions of up to 10 million deaths per year by 2050 [3]. The rise in antimicrobial resistant bacteria, especially multidrug-resistant (MDR) bacteria, together with the failure to develop new antibiotics has motivated researcher aimed at the development of novel therapeutic strategies, including those based on new antimicrobial agents [4,5,6,7], such as antimicrobial peptides (AMPs) [8,9]. A number of AMPs are currently in use or under study in clinical trials as a viable option to overcome resistance, either in combination with or as an alternative to existing antibiotics [10]. AMPs are produced by most living organisms as natural defensive molecules that protect against pathogenic microorganisms [11]. Despite their small size (2–9 kDa), AMPs are versatile, due to their structural and physicochemical properties. Moreover, the sequence of AMPs facilitates their amphipathic nature [12,13]. Nearly all AMPs have a positive net charge that allows them to interact selectively with bacterial membranes or other negatively charged structures. In order to obtain new antibacterial molecules, we have investigated branched peptides which has led us to the synthesis of what we call super-cationic peptide dendrimers (SCPDs) that appear to be broad-spectrum antibacterial compounds acting on Gram-positive and Gram-negative bacteria. Nevertheless, time–kill kinetics and growth curves, revealed considerable differences in their action, showing higher activity against Gram-negative bacteria. Among a long series of molecules G1OLO-L_2_OL_2_ displayed excellent microbiological results. The most prominent characteristic of SCPDs is their number of positive charges.

The bacterial membrane mediates interactions with both other organisms and the environment and is a key factor in the development of drug resistance [14]. The physical and chemical properties of biological membranes are directly linked to their functions [15]. In this work, we investigated the membrane interactions of the branched AMP rich in Ornitine (OLO)_2_KLLOLL-NH_2_ (G1OLO-L_2_OL_2_), from the recently described super-cationic peptide dendrimers (SCPDS) family [16]. To do so, we monitored the steady-state fluorescence of the polarity-sensitive probe Laurdan, and the fluorescence anisotropy of liposome-incorporated fluorescent molecules as a function of temperature. Membrane-peptide interactions were followed using atomic force microscopy (AFM). 

## 2. Materials and Methods

### 2.1. Materials 

**Figure 1:**
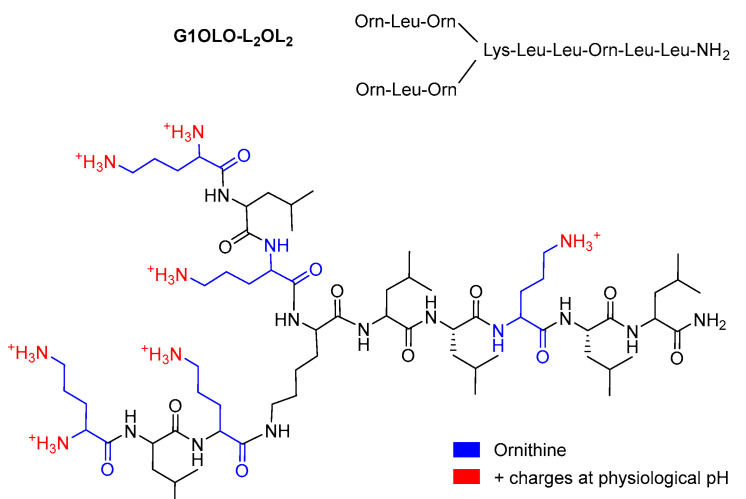
Chemical structure of the G1OLO-L_2_OL_2_ peptide.

Lipids 1-palmitoyl-2-oleoyl-*sn*-glycero-3-phosphoethanolamine (POPE) and 1-palmitoyl-2-oleoyl-*sn*-glycero-3-[phospho-rac-(1-glycerol)] (sodium salt) (POPG) were purchased from Avanti Polar Lipids (Alabaster, AL, USA) and dissolved in a chloroform:methanol (3:1,*v*/*v*) solution to a final concentration of 1 mg/mL. G1OLO-L_2_OL_2_ was synthesized as previously described [16]. Figure 1 shows the chemical structure of the G1OLO-L_2_OL_2_ peptide. The buffer used throughout the experiments was 10 mM Tris-HCl (pH 7.40) supplemented with 150 mM NaCl, prepared in ultrapure water (Milli-Q reverse osmosis system, 18.2 mΩ·cm resistivity). 1,6-Diphenyl-1,3,5-hexatriene (DPH), 1-(4-trimethylammoniumphenyl)-6-phenyl1,3,5-hexatriene p-toluenesulfonate (TMA-DPH), 6-dodecanoyl-2-dimethylaminonaphthalene (Laurdan), and 1-anilinonaphthalene-8-sulfonic acid (ANS) were purchased from Thermo Fisher Scientific, Inc. (Waltham, MA, USA). G1OLO-L_2_OL_2_ lyophilized peptide was fully dissolved in ultrapure water supplemented with 5% acetic acid to a final concentration of 5 mM. Volumes from this stock solution were used for all the experiments. Concentration in experiments was 0.005 µM but for AFM of SLB (0.0005 µM).

### 2.2. Bacterial Strains

Two imipenem-resistant *Pseudomonas aeruginosa* isolates (846VH, 536SJD) and, as a quality control, one collection strain of *P. aeruginosa* (ATCC 27853) were studied. For the AFM bacterial studies, *Escherichia coli* strain ATCC 25922 was also used. All isolates were stored as tryptic soy broth (TSB)-glycerol (15%) stocks at −80 °C and subcultured for use in the experiments.

### 2.3. Liposome Preparation 

Liposomes were prepared as follows: the corresponding volume of each phospholipid was prepared in a conical glass tube as described above. The solvent was then evaporated under a stream of oxygen-free N_2_ during constant rotation of the tube. The tube was kept under a vacuum overnight and protected from light. The dry lipid film was then resuspended in buffer to a final concentration of 500 µM. Multilamellar vesicles (MLVs) were formed after several freeze–thaw cycles below and above the transition temperature of the lipids (22 °C). The MLVs were then extruded through an Avanti^®^ Mini-extruder (Avanti Polar Lipids, Inc.), using polycarbonate membranes with a pore size of 100 nm.

The mean particle size and polydispersity of the liposomes were measured by dynamic light scattering, using a Nanosizer Nano S (Malvern Instruments, Malvern, UK). Electrophoretic mobility, indicating the effective surface electrical charge (potential), was determined using a Zetasizer Nano ZS90 (Malvern Instruments, UK). Each sample was measured in triplicate.

### 2.4. Electrostatic Surface Membrane Potential (∆Ψ) Measurements

The interaction of liposomes with macromolecules, including peptides, will depend on the balance of electrostatic repulsion vs. attraction forces and thus on the surface charge of the structures involved. In this study, the surface charge of the liposomes in the presence or absence of G1OLO-L_2_OL_2_, was determined using ANS, a negatively charged fluorescent probe with a low fluorescence yield in polar environments. Liposomes (500 µM) in the presence or absence of G1OLO-L_2_OL_2_ (incubated under the same conditions as in the DPH experiments) were titrated with 5 mM ANS in methanol and fluorescence was monitored at λ_ex_ and λ_em_ of 380 and 480 nm, respectively, using a lipid:probe ratio of 300/1 (mol/mol). The concentration of bound ANS ([*ANS*]*_B_*) vs. free ANS ([*ANS*]*_free_*) was adjusted using a modified Langmuir isotherm (Equation (1)):

(1) $${\left[ANS\right]}_{B}=C_{max}\frac{{\left(k\cdot {\left[ANS\right]}_{free}\right)}^{b}}{1+{\left(k\cdot {\left[ANS\right]}_{free}\right)}^{b}}$$

where *C_max_* is the maximum concentration of ANS bound to the liposomes, *k* is the binding constant, and *b* is a parameter value related to the cooperativity of the process. 

[*ANS*]*_B_* values can be calculated as shown in Equation (2):

(2) $${\left[ANS\right]}_{B}=\frac{F_{b}-F_{0}}{A_{b}-A_{0}}$$

where *F_b_* and *F*_0_ are the fluorescence intensities, and *A_b_* and *A*_0_ are the emission coefficients of ANS in the presence or absence of lipid, respectively. Emission coefficient *A_b_* can be evaluated as the slope of a high-lipid-concentration sample (2 mM) titrated with a diluted ANS solution (0.1–1 µM), and *A*_0_ from the same slope in the absence of lipid.

The change in the electrostatic surface potential (ΔΨ) of the liposomes due to incorporation of the peptide can be calculated as shown in Equation (3):

(3) $$\Delta \Psi =\frac{RT}{F}ln\left(\frac{k_{liposomes+peptide}}{k_{liposomes}}\right)$$

where *k_peptide_* and *k_liposomes_* are the ANS binding constants for liposomes in the presence or absence of the peptide, respectively, *R* is the universal gas constant, *T* is the absolute temperature, and *F* is Faraday’s constant.

All fluorescent determinations were carried out using an SLM-Aminco 8100 spectrofluorimeter equipped with a jacketed cuvette holder. The temperature was controlled (±0.1 °C) using a circulating water bath (Haake, Thermo Scientific, Waltham, MA, USA). The excitation and emission slits were 8/8 and 4/4, respectively.

### 2.5. Fluorescence Measurements

DPH tends to embed in the phospholipid bilayer, whereas TMA-DPH tends to anchor at its aqueous interface [17]. This difference was used to investigate the liposome phase behavior of the hydrocarbon domain of the bilayer. The liposomes were incubated with 0.005 µM of G1OLO-L_2_OL_2_ at 37 °C overnight. DPH or TMA-DPH was then added to the sample to a final lipid-to-probe ratio of 300/1 (mol:mol), followed by incubation for 30 min at 37 °C to allow the probe to interact with the liposomes. The anisotropy (*r*) of the samples over a temperature range of 3–45 °C was recorded at excitation and emission wavelengths of 381 nm and 526 nm, respectively. Vertically and horizontally polarized emission intensities were corrected for background scattering by subtracting the corresponding polarized intensities of a blank containing the unlabeled suspension (liposomes in buffer without the probe). The *r* values were calculated as shown in Equation (4):

(4) $$r=\frac{I_{VV}-G\cdot I_{VH}}{I_{VV}+2G\cdot I_{VH}}$$

where *I_ij_* is the fluorescence intensity when the excitation (*i*) and emission (*j*) polarizers are fixed in the vertical (*V*) or horizontal (*H*) position, and *G* is the instrument sensitivity ratio of the detection system for vertically and horizontally polarized light. 

The values of *r* as a function of temperature were adjusted using a modified Boltzmann equation (Equation (5)):

(5) $$r=r_{2}\frac{r_{1}-r_{2}}{1+10^{-b\left(\frac{T}{T_{m}}-1\right)}}$$

where *r*_1_ and *r*_2_ are the maximum and minimum values of *r*, *T_m_* is the *L*_β_-to-*L*_α_ phase transition temperature of the sample, and *b* is a parameter that provides information on the cooperativity of the transition process. 

Laurdan is a polarity-sensitive probe with an affinity for the glycerol backbone of the bilayer; its lauric acid tail anchors to the phospholipid acyl chain region [18]. In this study, Laurdan was used to monitor the bilayer fluidity related to a fluorescence shift, by taking advantage of its dipolar relaxation characteristics. Laurdan excitation was measured over a range of 320–420 nm, using emission wavelengths of 440 nm and 490 nm. The lipid concentration in the liposome suspension was adjusted to 250 μM, with Laurdan added to obtain a lipid:probe ratio of 300:1. The generalized polarization (*GP_ex_*) for the emission spectra was calculated as shown in Equation (6)

(6) $$GP_{ex}=\frac{I_{440}-I_{490}}{I_{440}+I_{490}}$$

where *I*_440_ and *I*_490_ are the fluorescence intensities at emission wavelengths of 440 nm (gel phase, *L*_β_) and 490 nm (liquid crystalline phase *L*_α_), respectively. *GP_ex_* values depend on the excitation wavelength (λ_ex_). In lipid mixtures and at constant temperature, positive slope values of *GP_ex_* vs. λ_ex_ indicate the coexistence of domains of different composition, and negative slope values a thermal transition towards a more fluid phase.

### 2.6. Atomic Force Microscopy Imaging 

#### 2.6.1. Bacteria 

The in vivo effects of G1OLO-L_2_OL_2_ onto the surfaces of *Pseudomonas* strain 27,853 and *E. coli* strain ATCC 25,922 after 4 h of exposure to the peptide at concentrations of 0.02 μM (2 × MIC) and 0.04 μM (4 × MIC) and 0.01 μM (2 × MIC) and 0.02 μM (4 × MIC), respectively, were assessed using AFM. Both strains were grown on Muller-Hinton broth cation-adjusted (MHBCA) medium to a concentration of 10^6^ colony-forming units (CFU)/mL. After incubation of the bacteria in fresh medium at 37 °C for 24 h to obtain cultures in the exponential growth phase, G1OLO-L_2_OL_2_ was added to the cultures for 4 h at an incubation temperature of 37 °C. The cells were then harvested by centrifugation, resuspended in 2% glutaraldehyde in 0.2 M PBS overnight, and washed three times with distilled water to remove cell debris. The pellets were then suspended in 1.5 mL of distilled water. A 10 μL drop of the suspension was placed on a Thermanox^®^ coverslip and glued to a mica disc for AFM imaging.

The samples were imaged in air using an atomic force microscope XE-70 (Park Systems, Suwon, Korea). Images were obtained in non-contact mode using pyramidal-shaped silicon cantilevers with a spring constant of ±40 N/m and a resonance frequency of ±300 kHz; the upper sides were coated with aluminum to enhance the reflectivity of the laser beam. AFM images were acquired with a scan size of 5 μm^2^ at a scan rate of 0.3–0.6 Hz. Data acquired during surface scanning were converted into images of topography and amplitude and analyzed using XEP and XEI software (Park Systems, Korea). The topography images were then used to observe the shape, structure, and surface of the planktonic bacteria. In addition, they were used to determine the average surface nano-roughness (*R*_a_) of the treated and untreated planktonic bacteria, with (*R*_a_) calculated as the average distance from the roughness profile to the center plane of the profile. 

#### 2.6.2. Liposomes

AFM was carried out on a commercial multimode atomic force microscope controlled by Nanoscope V electronics (Bruker AXS Corp., Madison, WI, USA). Freshly cleaved mica discs (1 cm^2^) mounted on round Teflon discs were glued to steel discs. Liposome suspensions were incubated overnight on the mica discs at 37 °C. To prevent sample evaporation, the steel discs containing the mica discs and the sample were enclosed in a small Petri dish placed inside a larger Petri dish with a small amount of water at the bottom as a reservoir. The large Petri dish was then sealed with Teflon ribbon and placed inside an oven (Termaks AS, Bergen, Norway) for 20 min with a temperature control of ±0.2 °C. Non-adsorbed liposomes were removed by gently rinsing the samples with buffer, covering the mica surface with 60 µL of buffer. The samples were then directly mounted on the AFM scanner (“E” scanner, 10 µm) and allowed to stabilize. Images were acquired in liquid using MSNL-10 sharpened silicon nitride tips (Bruker AXS Probes, Camarillo, CA, USA) with a mean spring constant of 30 pN nm^−1^, in contact mode at a 0° scan angle, and with a scan rate of 1.5 Hz. To minimize the applied force on the sample, the set point was continuously adjusted during imaging. Peptide was injected to a final concentration of 5 nM. All images were processed using NanoScope analysis software (Bruker AXS Corp., Santa Barbara, CA, USA).

### 2.7. Synergy Study

Checkerboard testing was used to assess the susceptibility of the planktonic, imipenem-resistant bacteria (*P. aeruginosa* strains 846VH and 536SJD) to G1OLO-L_2_OL_2_, added in combination with imipenem. Bacteria at a concentration of 10^6^ CFU/mL were added together with G1OLO-L_2_OL_2_ in imipenem-containing MHB (pH of 7.3 ± 0.2) to the wells of a 96-well round-bottom microtiter plate. Concentrations assayed were between 0.5 µg/mL to 16 µg/mL (Imipenem) and between 0.125 µg/mL to 64 µg/mL of the peptide. All experiments were performed in triplicate. The interactions of the bacteria with the peptide were quantitatively evaluated by calculating the fractional inhibitory concentration index (FICi) according to the following formula: FICi = ([MIC drug X in combination)/(MIC of drug X alone])+([MIC of drug Y in combination)/(MIC of drug Y alone]). An FICi < 0.5 was considered to indicate a synergistic interaction, an FICi > 4 an antagonistic interaction, and an FICi ≥ 0.5 and ≤ 4 an indifferent interaction [19].

## 3. Results

### 3.1. Particle Size and Z Potential

The size, polydispersity index (PDI), and zeta potential of the liposome and peptide are summarized in Table 1. The peptide was positively charged at the pH studied and, when incubated with the liposomes, did not significantly modify their size. Interestingly, the peptide did increase the zeta potential of blank liposomes by nearly 5 mV, from −31 mV to −26 mV.

### 3.2. Fluorescence Experiments

**Figure 2:**
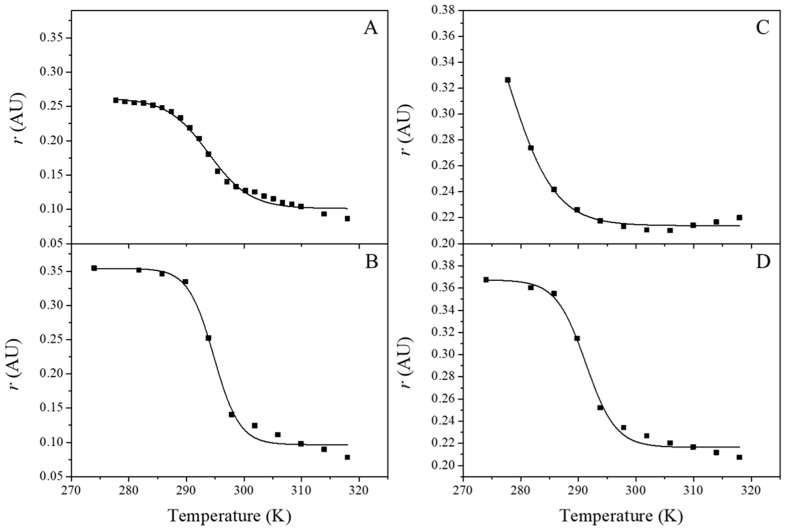
Temperature dependence of the fluorescence anisotropy (*r*) of POPE:POPG (3:1, mol/mol) liposomes incubated with DPH (**A**,**B**) or TMA-DPH (**C**,**D**). (**A**,**C**) Blank liposomes; (**B**,**D**) liposomes incubated with 5 nM G1OLO-L_2_OL_2_.

Changes in fluidity due to the presence of the peptide in the lipid membrane were analyzed based on the fluorescence anisotropy of the membranes when incubated with the probes DPH and TMA-DPH. Figure 2 shows the changes in DPH and TMA-DPH anisotropy (r) as a function of temperature, both for blank liposomes (Figure 2A,C) and liposomes incubated with 5 nM G1OLO-L_2_OL_2_ (Figure 2B,D). 

According to Equation (2), there were no significant changes in the transition temperature (*T*_m_) of the hydrophobic region (DPH) of the liposomes following their incubation with the peptide (Table 2), whereas with TMA-DPH there was a significant shift towards higher temperatures. For both fluorescent probes, the presence of the peptide increased the cooperativity of the gel (*L_β_*) to liquid-crystalline (*L_α_*) phase transition. At low temperatures, the anisotropy values of DPH were higher in the liposomes in the presence of the peptide than in blank liposomes and very similar to those of TMA-DPH in the liposomes.

**Figure 3:**
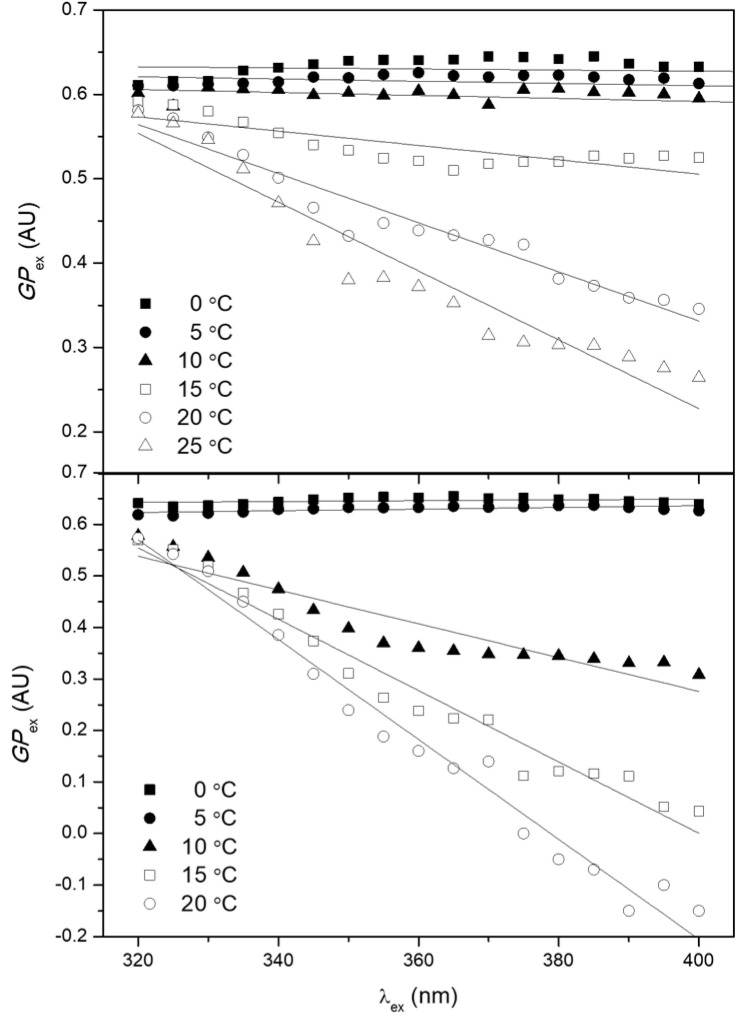
GP_ex_ values as a function of the λ_ex_ for POPE:POPG (3:1, mol/mol) liposomes at different temperatures. (**Top**) blank liposomes; (**Bottom**) liposomes incubated with 5 nM G1OLO-L_2_OL_2_.

The polarity-sensitive fluorescent probe Laurdan localizes to the glycerol backbone of a lipid bilayer, with its lauric acid tail anchored in the phospholipid acyl chain region. Laurdan is sensitive to the nature of the fluid phase of its lipid environment and is thus used to differentiate lipid microdomains differing in their lipid composition and lipid phase. In this study, Laurdan was used to analyze the induction of lipid microdomains in liposomes exposed to G1OLO-L_2_OL_2_. Figure 3 shows the changes in GP_ex_ as a function of λ_ex_ for the blank (Figure 3 Top) and for liposomes incubated with 5 nM G1OLO-L_2_OL_2_ (Figure 3 Bottom). As the λ_ex_ increased, GP_ex_ decreased at all temperatures studied, indicating a transition towards a more fluid phase for lipid domains that did not differ in their composition.

**Figure 4:**
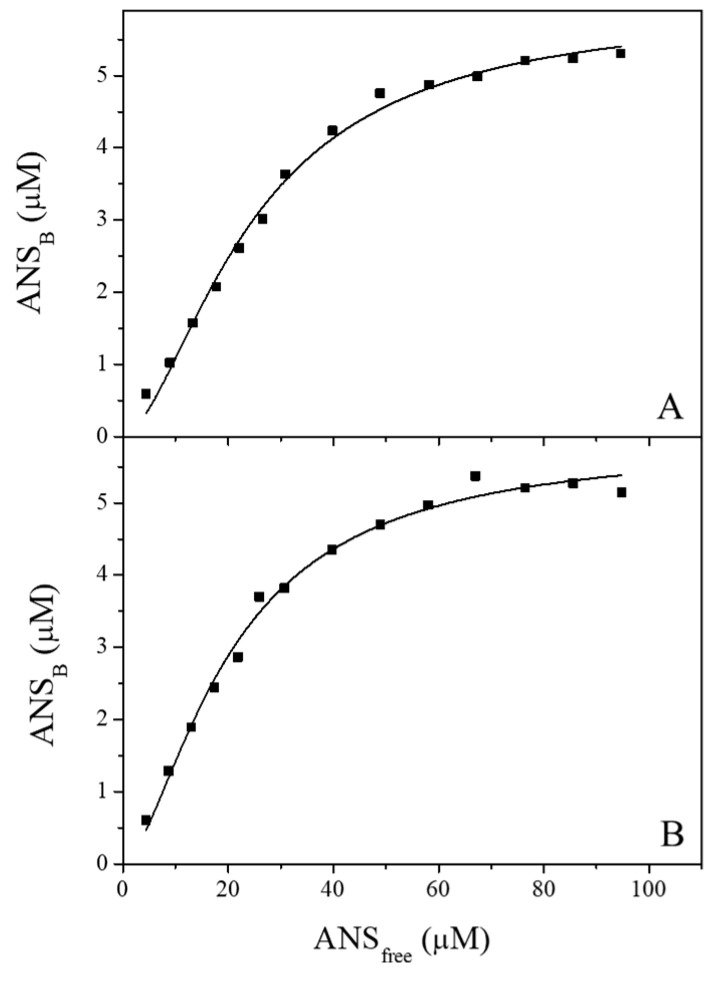
Concentration of ANS bound ([ANS]_B_) to POPE:POPG (3:1, mol/mol) liposomes as a function of the free ANS concentration ([ANS]_free_). (**A**) Blank liposomes; (**B**) liposomes incubated with 5 nM of G1OLO-L_2_OL_2_.

The changes in the zeta potential indicated that the peptide modifies the effective surface charge of the liposome. The zeta potential is the effective charge at the shear plane between the liposome and the medium. Since the peptide reaches the lipid bilayer surface, we examined the electrostatic modification of the surface potential of the liposomes using the probe ANS. For both the blank liposomes and the liposomes incubated with 5 nM G1OLO-L_2_OL_2_, titration of the sample with ANS increased the fluorescence signal. The amount of ANS bound to the liposome (ANS_B_) was calculated using Equation (5). In Figure 4, this value is represented as a function of the free ANS concentration in the presence of blank liposomes (Figure 4A) and liposomes incubated with 5 nM G1OLO-L_2_OL_2_ (Figure 4B). 

Fitting the data to Equation (4) (Table 3) yielded two very similar curves (C_max_ and b were not significantly different) but the binding constant was higher in the presence of G1OLO-L_2_OL_2_. According to Equation (6), in the presence of the peptide (5 nM) the surface potential of the blank liposomes increased by 5.06 mV.

### 3.3. Atomic Force Microscopy Studies

**Figure 5:**
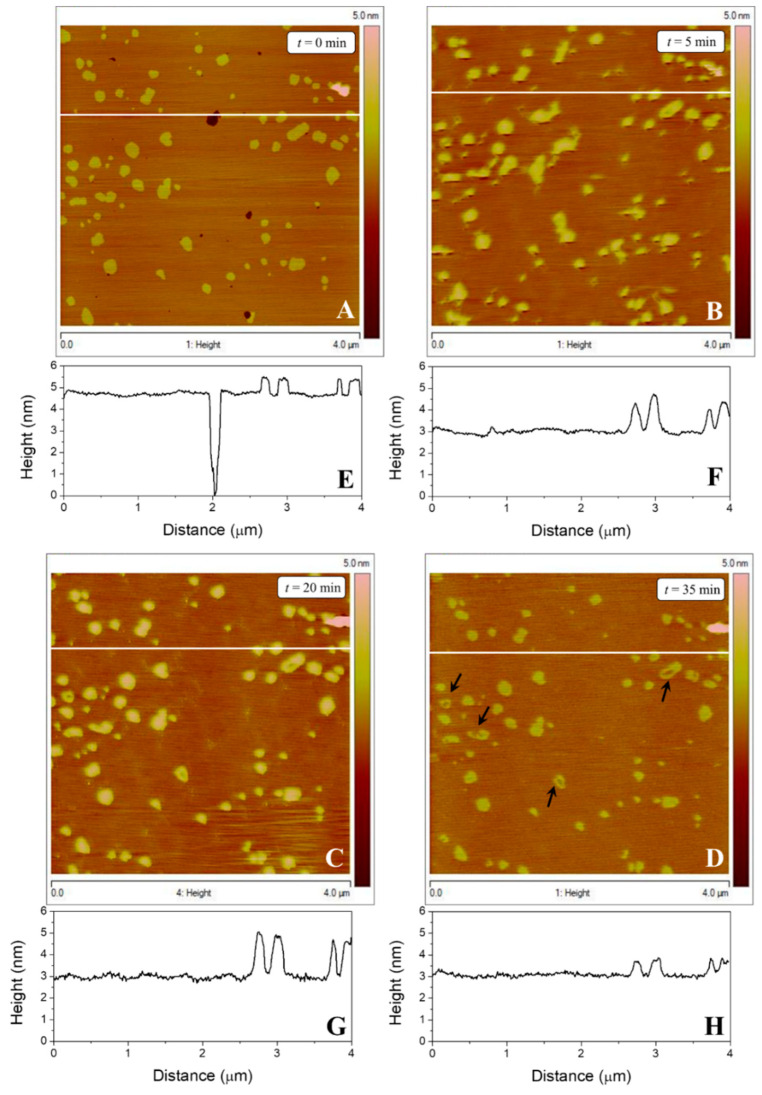
AFM topographic images of a SLB of POPE:POPG (3:1, mol/mol) incubated with 5 nM G1OLO-L_2_OL_2_ for different incubation times (**A**–**D**). Subfigures show one line topography profile for each image (**E**–**H**) represent the sample height along the white line in the corresponding AFM topographic image. Black arrows in (**D**) point to the degradation of the lipid domain.

Supported lipid bilayers: AFM has been used extensively to visualize the effect of small and large molecules on model lipid membranes. Although individual peptides are too small to visualize directly, their effects on lipid membranes can be followed at a subnanometer resolution using AFM. Figure 5 shows the AFM images (Figure 5A–D) and height line profiles (Figure 5E–H) of a supported lipid bilayer (SLB) of POPE:POPG (3:1, mol/mol) onto the mica surface. The SLB prior to its incubation with the peptide is shown in Figure 5A. From the image, three different regions can be defined: (i) small dark patches, attributable to the uncovered mica surface; (ii) a wide extended region (orange) with a step height difference of 4.61 ± 0.18 nm over the mica surface; and (iii) small yellowish domains protruding 0.66 ± 0.05 nm from the extended region, with a step height of 5.27 nm from the mica surface to the top of the yellowish domains. The heights of the different regions are depicted in the profile line in Figure 5E and were determined along the white line in Figure 5A. Five minutes after injection of the peptide to a final concentration of 5 nM (Figure 5B), the mica surface was fully covered, evidenced by the disappearance of the dark patches seen in Figure 5A, presumably due to fluidification and high lateral mobility of the lower lipid layer. The blurry borders of the higher lipid domains were probably due to the short stabilization time after injection of the peptide into the AFM cell. Following peptide injection, the high lipid domains protruded 1.56 ± 0.15 nm from the lower lipid domain but assumed a shape similar to that seen in Figure 5A. After 20 min. (Figure 5C) the lipid borders of the high lipid domains were well defined, no lipid domain appeared or vanished, and the step height from the bottom of the lower lipid domains to the top of the higher lipid domains was 1.84 ± 0.16 nm. After 35 min (Figure 5D), the step height difference between the lipid domains was 0.74 ± 0.06 nm. However, although high lipid domains retained their shape after peptide addition, areas indicating their degradation (black arrows) were also observed. A similar degradation occurred at shorter incubation times. In both cases, the lipid domains lost their structure, disorganizing from the center of the domain to the margins.

**Figure 6:**
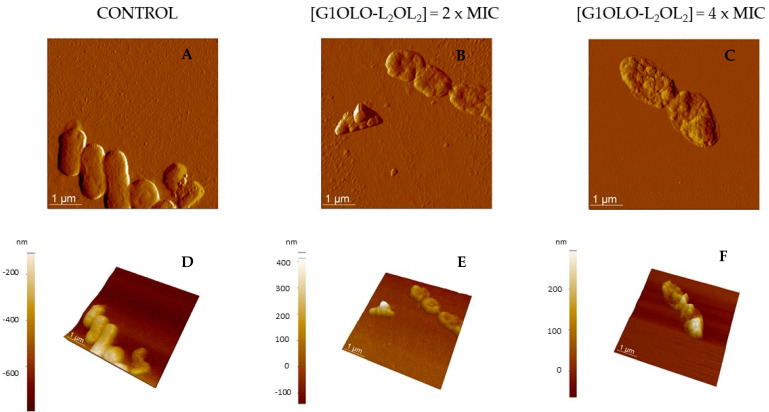
AFM amplitude (**A**–**C**) and 3D topographic images (**D**–**F**) of *P. aeruginosa* (27,853) incubated with different concentrations of the peptide G1OLO-L_2_OL_2_.

Bacteria: AFM was also used to investigate the effect of G1OLO-L_2_OL_2_ on planktonic cultures of *P. aeruginosa* (27,853) and *E. coli* (ATCC 25,922). Figure 6 shows the amplitude (Figure 6A–C) and 3D height (Figure 6D–F) of *P. aeruginosa* colonies incubated with different concentrations of G1OLO-L_2_OL_2_, as determined by AFM. In the control images (Figure 6A,D), the rod-shaped cells adsorbed onto the mica have flat and smooth surfaces, whereas after incubation with 0.02 µM G1OLO-L_2_OL_2_ (2 × MIC) (Figure 6B,E) the bacterial surface was characterized by a cluster of small round structures 200–300 nm wide. Many of the round structures were as tall as the original bacterium, with the most peripheral being those with a lower step height from the mica surface. A higher G1OLO-L_2_OL_2_ peptide concentration (4 × MIC) (Figure 6C,F) destroyed the bacterial cells. Roughness (R_a_) values can be found in Table 4 where the addition of the peptide increased the R_a_ value of the bacteria. 

**Figure 7:**
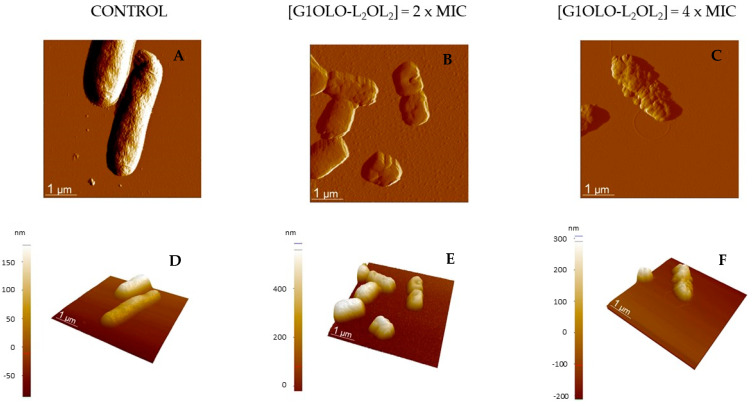
AFM amplitude (**A**–**C**) and 3D topographic (**D**–**F**) images of *E. coli* (ATCC 25,922) incubated with different concentrations of G1OLO-L_2_OL_2_.

Similar studies were performed on *E. coli.* Figure 7 shows the AFM amplitude (Figure 7A–C) and 3D height (Figure 7D–F) of *E. coli* in the absence or presence of G1OLO-L_2_OL_2_ at different concentrations. In the absence of the peptide, *E. coli* assumed their typical rod-shaped structure, with each cell having a length of 1–2 μm. However, incubation of the cells with 0.01 µM G1OLO-L_2_OL_2_ (2× MIC) (Figure 7B,E) resulted in a considerable reduction in cell size and a slight modification of the cell surface. At a concentration of 0.02 µM G1OLO-L_2_OL_2_ (4 × MIC) (Figure 7C,F), the shape of the bacteria was largely maintained but many small round structures appeared over the cell surface. Nano-roughness values of bacterial surface (treated and untreated) are reported in Table 4, showing a large increase in the initial values of Ra when increasing the peptide concentration.

### 3.4. Synergy Study

A possible synergistic effect of G1OLO-L_2_OL_2_ and imipenem on imipenem-resistant *P. aeruginosa* was examined to determine the effect of the peptide on the bacterial membrane. Specifically, we determined whether G1OLO-L_2_OL_2_ acted as a pore opener or disturbed membrane integrity, such that imipenem was then able to penetrate the bacteria, with lethal consequences. The FICi values obtained from the synergy studies are shown in Table 5. The values of FICi obtained in the checkerboard analysis of the potential synergy between G1OLO-L_2_OL_2_ and imipenem in MDR (imipenem-resistant) *P. aeruginosa* demonstrate indifference which is consistent with the biophysical data. Together, these results show that, while G1OLO-L_2_OL_2_ alters the bacterial outer membrane, it is unable to open channels or induce membrane discontinuities that would allow imipenem to penetrate imipenem-resistant *P. aeruginosa*.

## 4. Discussion

In a previous work [16] we described a new AMP family, grouped under the name super-cationic peptide dendrimers (SCPDs). Although all members exert antibacterial activity, some of them were shown to be selective for Gram-negative species but with virtually no cytotoxicity in HepG2 and HEK293 human cells. These results suggested that SCPD peptides could serve as a valuable class of AMPs. Among the SCPDs, G1OLO-L_2_OL_2_ was one of the most promising candidates for the development of an antibacterial agent against Gram-negative bacteria. The most prominent difference between Gram-negative and Gram-positive bacteria is the presence in the former of an outer membrane that acts as a permeability barrier—although the mechanism that ultimately kills bacteria may act on the internal (cytoplasmic) membrane. Our final aim was to decipher the mechanisms by which G1OLO-L_2_OL_2_ is able to kill Gram-negative MDR and, particularly in this work we aimed to gain insights on its interaction with model membranes and living cells. Using biophysical approaches, we examined the interaction of G1OLO-L_2_OL_2_ with model membranes mimicking the inner membrane of *E. coli* (POPE:POPG 3:1, mol/mol) [19]. When G1OLO-L_2_OL_2_ is incubated with liposomes in suspension, it preferentially interacts with the latter’s phospholipid head groups, as can be seen by the shift towards a higher melting transition temperature detected with the TMA-DPH probe. Something that does not occur with the DPH probe that resides in the core of the bilayer. This indicated an increase in the rigidity of the headgroup region of the liposome due to its interaction with the peptide. In their interactions with liposomes, peptides can either be adsorbed onto the membrane or be partially absorbed into the lipid-water interface, close to the upper portion of the fatty acyl chains, where TMA-DPH tends to localize. However, it cannot be excluded that this increase in the Tm, might be attributed to a charge screening selective effect due to the interaction of the peptide with the negatively charged POPG. The fluorescence results showed that the peptides were not found in the hydrophobic core region of the liposome, although their ability to permeate the membrane by forming pores cannot be excluded. In spite of the structural differences, a similar behavior was observed for a series of three nine residue peptides that contained unnatural amino acids in the primary sequence [20]. Nevertheless, the addition of G1OLO-L_2_OL_2_ to reconstituted black lipid bilayers did not generate noticeable electrophysiological phenomena, thus suggesting that the peptide was unable to generate true transmembrane channels (data not shown). 

In the AFM experiments, G1OLO-L_2_OL_2_ was injected in situ inside the AFM liquid cell at the same concentration used with liposomes in solution. In the absence of injected peptide, the AFM images revealed two lipid domains in the SLB surfaces, in concordance with our previous study [21]. Since the Laurdan fluorescence provided no evidence of the existence of domains of different lipid composition on the liposomes, these lipid domains may have been: (i) domains induced by changes in temperature of the same lipid composition, in which lipids in the taller domains were in a more rigid phase than those in the shorter domains or (ii) domains of different lipid composition in a different lipid phase, in which the presence of the mica surface decreased the lateral diffusion of the lipids, thus promoting the formation of segregated lipid domains differing in their lipid composition. The work performed in this study and in previously published work [22] indicated that the taller lipid domains were POPG enriched, and the more extended domains POPE enriched.

**Figure 8:**
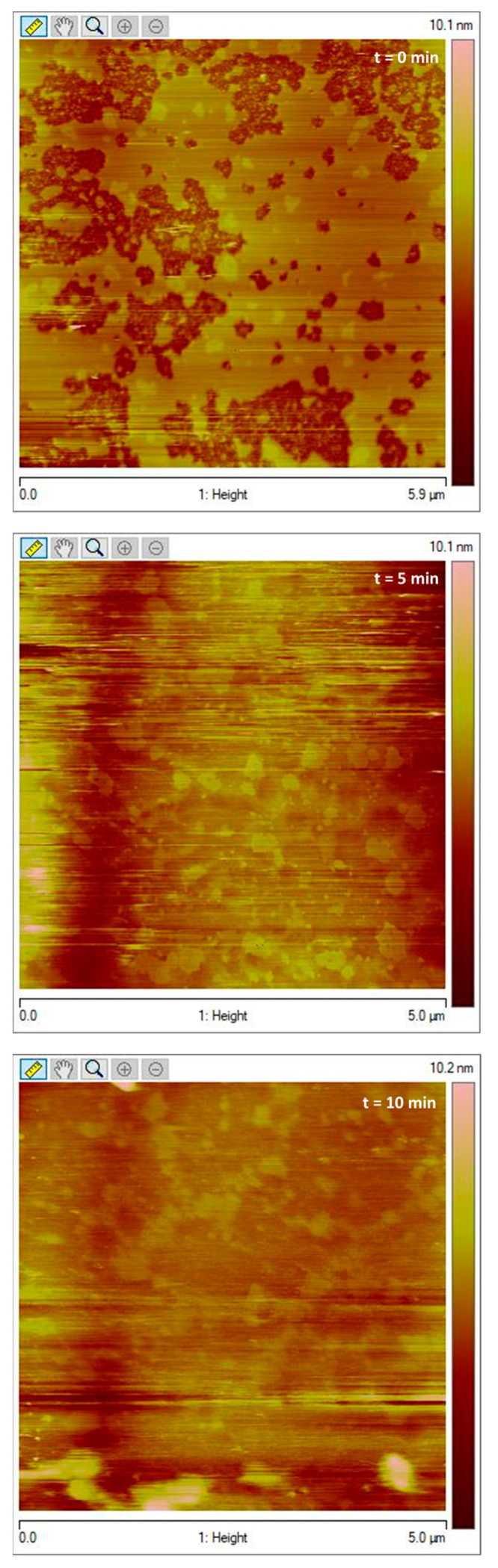
Erosion and solubilization of the SLBs at high peptide concentrations.

When added to the SLBs, G1OLO-L_2_OL_2_ interacted with their surfaces, without formation of pores, in agreement with the synergy studies. According to these observations, the peptide, at the concentrations studied, was able to adsorb onto or be partially absorb into the surface of the SLBs. However, a dose-dependent effect of the peptide was also observed, as higher concentrations (Figure 8) induced the erosion and solubilization of the SLBs. A similar effect was observed in the fluorescence experiments (data not shown), in which higher G1OLO-L_2_OL_2_ concentrations induced the erratic behavior of the liposomes, most likely attributable to their destabilization.

In agreement with these observations, determinations of the zeta potential of the liposomes were consistent with the incorporation of G1OLO-L_2_OL_2_ into the vesicles. However, to confirm that the peptide was in close contact with the lipid membrane of the liposome and not located in the hydration layer (where the zeta potential is actually measured), the surface potential was determined in an ANS fluorescence assay, which also showed that the peptide was present on the liposome surface.

Finally, the effects of G1OLO-L_2_OL_2_ on living bacteria were evaluated by AFM. The peptide had a more destructive effect on *P. aeruginosa* than on *E. coli*. While at 2 × MIC *P. aeruginosa* was destroyed, while *E. coli* retained its shape and cell integrity to a certain degree. However, it should be noted that AFM reveals only the topography of the bacterial surface, not bacterial viability. It is therefore possible that the bactericidal effect was similar in *P. aeruginosa* and *E. coli*, but the destruction of the lipid outer membrane differed. Studies of the differences in the membrane lipid composition in the two species in model membranes could help to explain the differences in the observed behaviors. In fact, although membrane permeabilization is the main mechanism of action of AMPs against pathogens, additional mechanisms have been described in detail. This includes membrane destabilization, inhibition of macromolecular synthesis and intracellular translocation and inhibition of the biosynthesis of nucleic acids and proteins [13]. The bacterial cytoplasm possesses a high osmotic potential that is maintained by the function of bacterial envelopes. The alteration of the membrane and/or the cell wall may determine a water influx and generate hydrostatic pressures incompatible with bacterial growth and even with survival. This is known as osmotic stress and has in bacteria some characteristics clearly different from those in the eukaryotic cells [23]. Here, the presence of low concentrations of G1OLO-L_2_OL_2_ at the outer membrane surface could induce osmotic stress and thereby facilitate a destabilization of cell integrity at higher peptide concentrations. In spite of this, the clear effect of G1OLO-L_2_OL_2_ on bacterial membranes, and its action on other targets cannot be ruled out and should be further investigated.

## 5. Conclusions

This study evaluated the interaction of the SCPD peptide G1OLO-L_2_OL_2_ with model membranes, liposomes, and SLBs using biophysical and microbiological approaches. The results consistently pointed to a surface effect of G1OLO-L_2_OL_2_ on the model lipid membranes, not only the adsorption or a close proximity of the peptide to the surface, but to some extent its association with or absorption into the more hydrophilic region of the phospholipids. 

## Figures and Tables

**Table 1:** Liposome and peptide characteristics: size, PDI, and zeta potential. Measures were made by triplicate.

	Diameter (nm)	PDI	Zeta Potential ± SE (mV)
G1OLO-L_2_OL_2_	-	-	+5.6 ± 0.4
Blank liposomes	62.09	0.061	−30.8 ± 0.8
Liposomes + G1OLO-L_2_OL_2_	70.07	0.108	−26 ± 2

**Table 2:** Values of the experimental parameters obtained by fitting the data in Figure 2 to Equation (2). Measures were made by triplicate.

	POPE:POPG (3:1, mol/mol) Liposomes			
	DPH		TMA-DPH	
	Blank	+G1OLO-L_2_OL_2_	Blank	+G1OLO-L_2_OL_2_
A_1_ ± SE	0.2624 ± 0.004	0.354 ± 0.007	0.44 ± 0.09	0.367 ± 0.005
A_2_ ± SE	0.1011 ± 0.003	0.097 ± 0.006	0.214 ± 0.002	0.216 ± 0.003
*T*_m_ ± SE (K)	293.9 ± 0.4	294.8 ± 0.4	277 ± 3	291.3 ± 0.4
b ± SE	36 ± 3	57 ± 9	30 ± 7	49 ± 7
r^2^	0.991	0.990	0.991	0.991

**Table 3:** Values of the experimental parameters based on the ANS binding data using Equation (4).

	POPE:POPG (3:1, mol/mol) Liposomes	
	Blank	+G1OLO-L_2_OL_2_
*C* (µM)	6.0 ± 0.2	5.8 ± 0.2
*k* (µM^−1^)	0.040 ± 0.002	0.049 ± 0.003
*b*	1.67 ± 0.13	1.60 ± 0.14
r^2^	0.993	0.990

**Table 4:** Nano-roughness (*Ra*) average values from Figure 6 and Figure 7.

	*Ra* (nm)		
	Control ± SE	2 × MIC (4 h) ± SE	4 × MIC (4 h) ± SE
*E. coli* ATCC (25,922)	7.37 ± 0.53	24.07 ± 2.64	27.40 ± 2.35
*P. aeruginosa* ATCC (27,853)	8.25 ± 0.69	23.39 ± 2.55	30.62 ± 2.89

**Table 5:** MIC and FICi data from the synergy study of G1OLO-L_2_OL_2_ and imipenem.

	MIC				FICi
	Imipenem		G1OLO-L_2_OL_2_		
	μg/mL	μM/L	μg/mL	μM/L	
PA 846 VH	16	0.053	>64	0.046	1.1875
PA 356 SJD	16	0.053	>64	0.046	1.1875

## Data Availability

Not applicable.

## Abbreviations

AMR: antimicrobial resistance; MDR: multidrug-resistant; AMPs: antimicrobial peptides; SCPDS: super-cationic peptide dendrimers; TMA-DPH: 1-(4-trimethylammoniumphenyl)-6-phenyl 1,3,5-hexatrienep-toluenesulfonate; DPH: 1,6-diphenyl-1,3,5-hexatriene; ANS: 1-anilino-8-naphthalene sulfonate; AFM: atomic force microscopy; POPE: 1-palmitoyl-2-oleoyl-sn-glycero-3-phosphoethanolamine; POPG: 1-palmitoyl-2-oleoyl-sn-glycero-3-[phospho-rac-(1-glycerol)]; TMA-DPH: 1,6-Diphenyl-1,3,5-hexatriene (DPH), 1-(4-trimethylammoniumphenyl)-6-phenyl1,3,5-hexatriene p-toluenesulfonate; ANS 1-anilinonaphthalene-8-sulfonic acid; MLVs: Multilamellar vesicles; MHBCA: Muller-Hinton broth cation-adjusted; CFU: colony-forming units; PBS: Phosphate Buffer Saline; FICi: fractional inhibitory concentration index; MIC: minimum inhibitory concentration; SLB: supported lipid bilayer; HepG2: human liver cancer cell line; HEK293: human embryonic kidney cells grown in tissue culture.
